# Transition of a community‐ and person‐centred design for providing healthcare services to gay and bisexual men and other men who have sex with men who engage in chemsex from a facility‐based setting to a community‐led setting in Taiwan

**DOI:** 10.1002/jia2.26188

**Published:** 2023-11-05

**Authors:** Carol Strong, Jing‐Hao Hsu, An‐Chun Chung, Meng‐Tzu Wu, Yi‐Hui Wu, Kuo‐Wei Lo, Su‐Ting Hsu, Nai‐Ying Ko

**Affiliations:** ^1^ Department of Public Health College of Medicine National Cheng Kung University Tainan Taiwan; ^2^ Taiwan Love and Hope Association Kaohsiung Taiwan; ^3^ Healing, Empowerment, Recovery of Chemsex (HERO) Health Center HÉROS Kaohsiung Taiwan; ^4^ Department of Community Psychiatry Kaohsiung Municipal Kai‐Syuan Psychiatric Hospital Kaohsiung Taiwan; ^5^ Department of Nursing College of Medicine National Cheng Kung University Tainan Taiwan

**Keywords:** health systems, gender, HIV care continuum, community, chemsex, PrEP

1

The decline of new HIV acquisitions in Taiwan can be attributed to a combination of rapidly increasing antiretroviral therapy (ART) coverage, the successful implementation of HIV self‐testing and the uptake of pre‐exposure prophylaxis (PrEP) [1]. With regard to HIV prevention, the key population in Taiwan, gay and bisexual men and other men who have sex with men (GBMSM), has faced new challenges in the past decade from two major issues, the first of which is chemsex behaviour, a form of sexualized use of drugs, such as methamphetamine, gamma hydroxybutyrate/gamma butyrolactone (GHB/GBL) and mephedrone, and needed harm reduction strategies [2]. The second issue is the relative centralization of the service delivery for PrEP medications, which persistently hinders the scale‐up of efforts at PrEP.

A model for the provision of integrated sexual health services, the Healing, Empowerment, Recovery of Chemsex (HERO) Health Center, was established in Taiwan in 2017 by a non‐governmental organization (NGO) with a decade‐long relationship with the local Lesbian, Gay, Bisexual, and Transgender (LGBT) community. HERO works from a regional hospital where it served as a one‐stop healthcare centre integrating services for chemsex care, mental healthcare and PrEP/PEP (post‐exposure prophylaxis) [3].

In May 2022, HERO transitioned from the original model to a new social enterprise model and was renamed HÉROS. It now functions independently in an urban neighbourhood that allows easy access to GBMSM, people who engage in chemsex, people with HIV and the general population. It incorporates a pharmacy, a clinic and a community health centre, offering a one‐stop, person‐centred destination providing comprehensive care, and preventive treatments through the provision of differentiated and simplified PrEP [4].

HÉROS provides a series of integrated mental health services for people who engage in chemsex, including a psychiatric outpatient clinic, individual psychological counselling and various support groups, including chemsex recovery groups. There are three major types of groups led by clinical psychologists geared towards the specific needs of people at the different stages of the recovery journey. First is the early relapse prevention group which is aimed at individuals who have the intention to reduce their use of chemsex. During sessions, the issues dealt with include identifying high‐risk contexts and cravings, and developing coping skills. The second group follows a 12‐step programme targeted at chemsex users who are at the action stage—those managing their chemsex behaviour with the help of coping mechanisms, but remain vulnerable to relapse. Finally, the interpersonal skills group focuses on discussions of intimacy among clients who have achieved maintenance of their chemsex behaviour by mastering certain coping mechanisms. The interventions employed with this group explore the past negative experiences of these men that are associated with emotional regulation and attachment style [5]. Among GBMSM who engaged in chemsex at HERO, 23% had been members of a recovery group and 17% had visited a mental health clinic at HERO [6]. A high frequency of substance use and living with HIV were significantly associated with being members of a chemsex recovery group, but age, income, education and employment status were not.

After less than 1 year since it moved into the community in 2022 (May 2022–March 2023), HÉROS clinic has received 1576 clients contributing 3600 visits. The majority were visits to the internal medicine clinic (74%). Regarding the HIV‐related services provided at HÉROS, 19 people received PEP, 167 were living with HIV and 61 people received ART at HÉROS clinic when others chose to go to other clinics or hospitals. Additionally, 115 people received PrEP, 69 of whom were able to take advantage of a government programme that subsidizes PrEP medication, while the other 46 people paid out of pocket. About 90% of clients receiving PrEP or ART at HÉROS are GBMSM. There are three transwomen and two transmen receiving ART, PrEP or hormone therapy at HÉROS. More than one‐fifth of the 1576 clients aged more than 50 years, while 10% of individuals receiving ART at HÉROS aged more than 50 years. Vaccines were given at HÉROS to 40 people for shingles, 45 for Human papillomavirus (HPV), 27 for hepatitis B, 17 for hepatitis A, 5 for pneumococcal pneumonia and 7 for influenza. Finally, in its first year of operating independently, HÉROS has conducted more than 40 group sessions, with the participation of 70 chemsex‐practicing GBMSM. GBMSM clients at HÉROS for the past year are more likely to be in the higher socio‐economic status than those at HERO. People with more economic resources might be more likely to be early adopters of this new model and it will take time for the diffusion effect to spread through the social system.

The transformation from HERO to HÉROS did not occur without a few glitches in response to which a number of solutions are currently being devised. In Table 1, the domains and constructs of the Consolidated Framework for Implementation Research (CFIR) are used to organize the determinants of the implementation of these solutions at HÉROS [7]. Table 1 also shows how the solutions correspond to the implementation strategies proposed by Powell et al. [8].

**Table 1:** HÉROS characteristics corresponding to the domains and constructs of the Consolidated Framework for Implementation Research (CFIR)

CFIR domain [7]	CFIR constructs [7]	Implementation determinants	Major challenges	Solutions	Solutions corresponding to Powell's implementation strategies [8]
**Intervention characteristics**	Relative advantage and adaptability	Efforts at community outreach based on working as an NGO since 1999 made it possible to attract members of the LGBT community and people who engage in chemsex to HÉROS. Recovery group sessions with a focus on HIV have been running since 2015 at the community health centre. The location in the midst of the community facilitates access.	The location of HÉROS may concern its neighbours because HIV stigma is prevalent in Taiwanese society.	We engaged local opinion leaders by holding townhall meetings where we explained the services we provide. At the pharmacy and clinic, we provide pamphlets, posters and handouts designed to raise the awareness of LGBT issues and to educate the general public about HIV and STIs.	Education strategies—Informing and influencing stakeholders; conducting educational outreach visits
**Outer setting**	Patient needs and resources	We provide a patient‐centred approach and a one‐stop integrated healthcare centre for people who engage in chemsex, including services for mental health, HIV/Sexually transmitted infections (STI) and substance use. The community‐based clinical setting enables a faster response to new infections, policies and interventions, such as monkey pox vaccination and long‐acting HIV treatment, compared to large hospitals.	People living with HIV and those who engage in chemsex are getting older like the general population. Therefore, preparing in advance for the needs of ageing care for people living with HIV and chemsex users, is a key element in transitioning to community care. There is an urgent need for incorporating chemsex care with other age‐related needs, such as long‐term care, social interaction and social support, and managing poor physical health and comorbidities associated with the ageing process.	The determination of which services have a more pressing need could be made based not only on the observations of healthcare providers, but also on feedback provided by clients through questionnaires filled out during each visit. Furthermore, the prediction and evaluation of the needs of the clients should be prioritized during the meetings of the community advisory board of HÉROS. With this in mind, the healthcare providers at the HÉROS clinic encourage our clients to consider getting vaccinated for shingles, pneumococcal pneumonia, influenza and so on. They also follow person‐centred protocols for providing services in order to ensure that gender‐diverse clients feel comfortable in visiting the centre for their medical and aesthetic needs. We invite fitness coaches to work with our therapists in order to design programmes for physical fitness and outdoor exercise as a way of improving the physical and mental health of our clients and of allowing them to maintain a degree of social contact. Finally, HÉROS pharmacists play the role of allies to our gender‐diverse clients by ensuring optimal health outcomes and by helping GBMSM achieve their therapeutic objectives.	Quality management strategies—Auditing and providing feedback; using advisory boards and work groups
**Inner setting**	Structural characteristics and culture	We provide multifaceted, integrated services with the help of several professionals, such as clinicians, pharmacists, therapists and social workers. The majority of the healthcare workers and staff at HÉROS identify as LGBT. The employees at HÉROS are selected based on their cultural competency and trained when it is inadequate.	Management across multiple sectors including a clinic, a pharmacy and a non‐profit organization can be difficult, but it is vital to the sustainability of the social enterprise model that HÉROS was built on.	Frequent meetings are held among the healthcare providers and other staff members to discuss the monitoring of the clinical process and the outcomes of the business model for each sector, and to review the budget and the electronically recorded data, which is important to identifying areas for improvement in the model.	Quality management strategies—Developing and organizing systems for monitoring quality

A few major challenges stand in the way of HÉROS functioning smoothly and effectively as an integrated sexual health services centre. First, being located amid a community facilitates access to sexual health services for chemsex‐practicing individuals; however, the location of HÉROS may concern its neighbours. HIV acquisition carries a stigma and discrimination against the LGBT community which is prevalent in Taiwanese society [9]. To address this, HÉROS exhibits a gender‐neutral attitude and welcomes both chemsex‐practicing LGBT community and non‐LGBT clients from the neighbourhood. For example, we made the extra effort of approaching local opinion leaders by holding townhall meetings where we explained the services we provide.

The second challenge is related to the fact that Taiwan is an ageing society. As we grow old, we all face difficulties such as receiving long‐term care, engaging in social interaction, finding social support, and managing poor physical health and comorbidities associated with the ageing process. This issue is compounded by the discrimination faced by the LGBT community in Taiwan, which highlights the need to incorporate chemsex care into existing healthcare frameworks. Furthermore, these efforts must aim to stay one step ahead by implementing health promotion plans before the ageing population exceeds the healthcare capacity. With this in mind, healthcare providers at HÉROS encourage our clients to consider getting vaccinated for shingles, pneumococcal pneumonia, influenza and so on. Finally, HÉROS pharmacists play the role of allies to our gender‐diverse clients by ensuring optimal health outcomes and by helping GBMSM achieve their therapeutic objectives [10].

The third sizeable obstacle of HÉROS, which may potentially jeopardize the sustainability of the social enterprise model upon which it was conceived, is the difficulty of simultaneously managing the work of multiple sectors, including a clinic, a pharmacy and a non‐profit organization. HÉROS emphasizes coming up with creative, innovative and enterprising ways of solving problems resulting from chemsex behaviour. The centre maintains its financial health by providing access to traditional services (e.g. counselling, prevention, diagnosis and treatment), as well as tweaking, modifying and hybridizing existing solutions/services for the LGBT community to produce novel techniques. HÉROS focuses not only on caring for clients with various gender identities and sexual orientations, but also on offering innovative services based on integrative strategies, such as home‐delivery packages and telemedicine. It is crucial to be able to identify areas for improvement in this model.

HÉROS employs a broad range of operational and ideological approaches to the planning and delivery of healthcare services to members of the community. However, there is room for improvement. It is essential to regularly assess and adjust the business model in order to ensure that it continues to meet the evolving needs of the community, especially in light of changing societal and political contexts. This ongoing process of evaluation and adaptation will enable HÉROS to continue providing high‐quality care and support to its clients.

## COMPETING INTERESTS

All authors have no competing interests to declare in relation to this work.

## AUTHORS’ CONTRIBUTIONS

CS, J‐HH and N‐YK wrote the original draft of the manuscript. N‐YK, CS, A‐CC and M‐TW were responsible for the research design, and they led the project. Y‐HW, K‐WL and S‐TH implemented the healthcare services. CS and N‐YK revised all drafts, which were reviewed and approved by all authors.

## FUNDING

This work received financial support from the National Science and Technology Council in Taiwan (grant numbers: 108–2636‐B‐006–004, 109–2636‐B‐006–004, 110–2636‐B‐006–011, 111‐2321‐B‐006‐009 and 111–2636‐B‐006–011), and Taiwan Centers for Disease Control (MOHW107‐CDC–C‐114–000107, MOHW108‐CDC–C‐114–000106 and MOHW112‐CDC–C‐114–000105).
